# Immune Correlates of Denileukin Diftitox Treatment in TFH-Type Lymphoma

**DOI:** 10.3390/cancers18101529

**Published:** 2026-05-09

**Authors:** Tatsuro Jo, Takahiro Sakai, Kazuhiro Noguchi, Kaori Yamaguchi, Kaho Umemoto, Masatoshi Matsuo, Yasushi Sawayama, Jun Taguchi, Ritsuko Kubota-Koketsu, Kuniko Abe, Kazuto Shigematsu

**Affiliations:** 1 Department of Hematology, Japanese Red Cross Nagasaki Genbaku Hospital, Mori-machi 3-15, Nagasaki City 852-8511, Nagasaki, Japan; 2 Department of Clinical Laboratory, Japanese Red Cross Nagasaki Genbaku Hospital, Mori-machi 3-15, Nagasaki City 852-8511, Nagasaki, Japan; 3 Specimen Analysis Team, Human Resource Development Division, Center for Infectious Disease Education and Research, The University of Osaka, Yamadaoka 2-2, Suita City 565-0871, Osaka, Japan; 4 Department of Pathology, Japanese Red Cross Nagasaki Genbaku Hospital, Mori-machi 3-15, Nagasaki City 852-8511, Nagasaki, Japan

**Keywords:** denileukin diftitox, TFH-type lymphoma, FOXP3-positive cells, tumor-associated macrophages, CD8-positive T-cells, T-cell receptor repertoire

## Abstract

**Background/Objectives:** Follicular helper T-cell (TFH)-type lymphomas, including angioimmunoblastic T-cell lymphoma (AITL) and TFH lymphoma, not otherwise specified (TFH-NOS), are characterized by marked immune dysregulation in the tumor microenvironment. We investigated whether TFH-type lymphomas are enriched in immunosuppressive cells and explored immunologic changes associated with denileukin diftitox (DD) treatment. **Methods:** FOXP3-positive mononuclear cells were quantified by immunohistochemistry in lymph node specimens from 10 patients with TFH-type lymphoma and six with non-TFH-type T-cell lymphoma. Paired skin biopsy specimens obtained before and after DD treatment from two patients with AITL were evaluated for CD4, CD8, CD68, CD163, and FOXP3 expression. Longitudinal flow cytometric T-cell receptor Vβ repertoire analysis of CD8-positive T-cell subsets was performed in three patients with AITL treated with DD. Clinical responses were retrospectively assessed in seven patients with relapsed or refractory TFH-type lymphoma treated with DD. **Results:** TFH-type lymphomas showed significantly higher intra-tumoral FOXP3-positive cell densities than non-TFH-type lymphomas (*p* = 0.0024). In paired skin biopsies, DD treatment was associated with reductions in CD68- and CD163-positive macrophage-rich infiltrates and a suggested decrease in FOXP3-positive cells. Among seven patients treated with DD, the overall response rate was 86%, including one complete response and five partial responses. Responders showed selective Vβ over-representation patterns in effector and/or memory CD8-positive T-cell subsets, whereas such findings were not convincing in the non-responder. **Conclusions:** TFH-type lymphomas were associated with higher FOXP3-positive cell density than selected non-TFH-type comparators. DD treatment may be associated with changes in tissue immune infiltrates and peripheral CD8/TCR Vβ skewing in responding cases, although these findings remain exploratory and do not establish causality.

## Simple Summary

Follicular helper T-cell lymphoma is a rare and aggressive type of T-cell lymphoma that often develops in a complex immune-rich tumor environment. In this study, we examined tumor samples and clinical data to explore immune features associated with denileukin diftitox treatment. We found that follicular helper T-cell lymphomas contained more FOXP3-positive cells than selected non-TFH-type T-cell lymphomas. In paired skin biopsy samples from two patients, post-treatment rash specimens showed reduced macrophage-associated staining and fewer FOXP3-positive cells compared with diagnostic skin lymphoma specimens. In addition, some responding patients showed skewed CD8-positive T-cell receptor Vβ patterns after treatment. These findings do not prove a causal mechanism, but suggest that denileukin diftitox treatment may be accompanied by changes in tissue infiltrates and peripheral CD8-positive T-cell profiles in some patients with follicular helper T-cell lymphoma.

## 1. Introduction

Angioimmunoblastic lymphoma (AITL) is a distinct entity within the spectrum of nodal T-cell lymphomas derived from follicular helper T (TFH) cells. In the recent WHO 5th edition classification and the International Consensus Classification (ICC), AITL and related nodal TFH-cell lymphomas are defined by shared clinicopathologic features, TFH immunophenotype, and characteristic genomic alterations, reflecting a unified disease biology centered on TFH lineage programs and immune dysregulation within the tumor microenvironment (TME) [1,2]. Clinically, AITL often presents with systemic symptoms and immune-mediated manifestations, and pathologically, it is characterized by a polymorphous infiltrate, prominent vascular proliferation, and expansion of follicular dendritic cell networks, underscoring the importance of the non-neoplastic immune compartment in disease behavior [3].

The TFH origin of AITL has been supported by gene-expression profiling and immunophenotypic studies demonstrating a molecular and functional link between AITL and TFH cells [4]. TFH-associated markers, including programmed cell death protein 1 and SLAM-associated protein, are frequently expressed by neoplastic T-cells in AITL and have been used to support diagnosis and disease classification [5]. Similarly, C-X-C motif chemokine ligand 13 is highly upregulated in germinal center-related helper T-cell programs and helps distinguish AITL from peripheral T-cell lymphoma, not otherwise specified (PTCL-NOS), particularly in diagnostically challenging cases [6]. Additional markers such as CD10 can be expressed by the neoplastic T-cell population and may aid in diagnosis; detection of CD10-positive T-cells by flow cytometry has been proposed as a practical adjunct in appropriate contexts [7,8]. Collectively, these observations emphasize that AITL is not solely a malignant T-cell proliferation, but a lymphoma in which the TFH program shapes the surrounding immune milieu.

At the molecular level, AITL and nodal TFH-cell lymphomas exhibit recurrent mutations affecting T-cell receptor signaling and epigenetic regulation. Ras homolog family member A mutations, particularly the G17V hot spot, are a hallmark of AITL biology and contribute to TFH-like transcriptional and signaling states [9]. Large-scale genomic studies have also highlighted additional recurrent alterations, including epigenetic regulators and signaling genes that converge on TFH differentiation and microenvironmental interactions [10]. Moreover, isocitrate dehydrogenase 2 mutations are enriched in AITL and are implicated in epigenetic dysregulation, providing a mechanistic bridge between genetic lesions and aberrant immune phenotypes within the TME [11]. These genomic features further support the concept that immune microenvironment remodeling is integral to AITL pathogenesis and may represent a tractable therapeutic axis.

Because AITL is defined by profound immune dysregulation, strategies that modulate suppressive immune subsets in the TME are of particular interest. Regulatory T-cells (Tregs) and immunosuppressive myeloid populations can attenuate antitumor cytotoxic T lymphocyte (CTL) responses, and therapeutic depletion of Tregs has been associated with enhancement of antitumor immunity in humans [12]. Denileukin diftitox (DD) is a fusion toxin targeting the interleukin-2 receptor (IL-2R) pathway and has demonstrated clinical activity in lymphoid malignancies, providing a precedent for immune-directed approaches that may alter suppressive cell populations and restore antitumor immune responses [13,14]. In Japan, the second-generation DD formulation (E7777) has been clinically evaluated in relapsed/refractory PTCL, further supporting feasibility and ongoing relevance of this immunotherapeutic concept in T-cell neoplasms [15].

In this context, detailed characterization of the AITL/TFH-lymphoma immune microenvironment and how it may be altered by immunomodulatory therapy remains clinically meaningful. In the present study, we (i) compared intratumoral FOXP3-positive cell density between TFH-type lymphomas and selected non-TFH-type T-cell lymphoma comparators, and (ii) explored tissue-based immune changes and peripheral CD8/T-cell receptor (TCR) Vβ repertoire patterns in patients with TFH-type lymphoma treated with DD. By integrating clinicopathologic data with exploratory immune profiling, we aimed to describe disease-specific immune correlates of DD treatment in TFH-type lymphoma.

## 2. Materials and Methods

### 2.1. Analysis of FOXP3-Positive Cells in Lymphoma Tissues

We analyzed lymph node biopsy specimens from 16 patients: 10 with TFH-type lymphomas (7 AITL and 3 TFH-NOS) and 6 with non-TFH-type lymphomas (2 PTCL-NOS, 1 Epstein–Barr virus-positive [EBV^+^] natural killer [NK]/T-cell lymphoma, 1 ALK-negative [ALK^−^] anaplastic large cell lymphoma [ALCL], and 2 adult T-cell leukemia/lymphoma [ATLL]) (Table 1). All lymph node specimens used for the FOXP3 analysis were diagnostic biopsy samples obtained before the initiation of systemic chemotherapy. Among the 10 TFH-type lymphoma cases included in the FOXP3 analysis, 6 cases overlapped with the DD-treated cohort shown in Table 2, excluding Pt-4. The remaining 4 TFH-type lymphoma cases, consisting of 2 AITL and 2 TFH-NOS cases, had not received DD therapy; these patients were treated with A-CHP, CHOP-like therapy in 2 cases, or EPOCH, respectively. Formalin-fixed, paraffin-embedded tissues were sectioned at 4 µm and subjected to immunohistochemistry (IHC) for FOXP3. FOXP3-positive mononuclear cells were counted as a surrogate marker of a putative regulatory T-cell-enriched compartment, but FOXP3 expression alone was not considered definitive proof of suppressive Treg identity. FOXP3-positive cell density was quantified as the number of FOXP3-positive cells per mm^2^, as previously described [16]. For each case, FOXP3-positive cells were counted in 15 intra-tumoral fields selected in a non-targeted manner to minimize intentional field-selection bias. Hotspot fields were not specifically targeted, but were not intentionally excluded if encountered during field selection. The mean number of FOXP3-positive cells across the 15 fields was used for analysis. Digital image analysis was not performed. Comparisons between the TFH-type and non-TFH-type groups were performed using the two-sided Mann–Whitney U test.

### 2.2. Immunohistochemical Evaluation of Macrophages and FOXP3-Positive Cells in Skin Biopsy Specimens

Skin biopsy specimens were analyzed from two patients with AITL who showed (i) cutaneous involvement by lymphoma cells at diagnosis and (ii) a subsequent skin rash clinically suspected to be related to DD therapy. Formalin-fixed, paraffin-embedded tissues were sectioned at 4 µm and stained with hematoxylin and eosin (HE). IHC was performed for CD4, CD8, CD68, CD163, and FOXP3 to evaluate lymphoid and macrophage populations. All antibodies for IHC were listed in Supplemental Table S1.

### 2.3. TCR Repertoire Analysis

Whole blood samples were collected at predefined or available time points during DD treatment and stained with fluorochrome-conjugated monoclonal antibodies and incubated for 10 min at room temperature. Erythrocytes were lysed using OptiLyse C Lysing Solution (Beckman Coulter, Brea, CA, USA) according to the manufacturer’s instructions. After lysis, cells were washed once with phosphate-buffered saline and resuspended for acquisition. No fixation was performed for these surface-staining panels.

CD8-positive T-cell subsets were defined phenotypically using CD27 and CD45RA, as previously described [17], and were operationally termed CTL subsets in this study:

- Naïve CD8-positive T-cells: CD8^+^CD27^+^CD45RA^+^;
- Effector CD8-positive T-cells: CD8^+^CD27^−^CD45RA^+^;
- Memory CD8-positive T-cells: CD8^+^CD27^+/−^CD45RA^−^.

For TCR Vβ repertoire analysis, lymphocytes were gated by forward scatter/side scatter, followed by gating on CD8^+^ events, and Vβ family frequencies were determined using IOTest Beta Mark TCR Vβ Repertoire Kit (Beckman Coulter, Brea, CA, USA), following the manufacturer’s protocol. Naïve/effector/memory subset frequencies were calculated as percentages of total CD8^+^ cells (CD8^+^ = 100%). Vβ family frequencies were calculated within each subset gate, i.e., within effector or memory CD8-positive T-cell subsets.

Operational categories of Vβ over-representation were defined as follows:

- Minor Vβ over-representation: 5−9.9%;
- Moderate Vβ over-representation: 10−14.9%;
- Dominant Vβ over-representation: ≥15% of a given Vβ family.

In this study, the term “CTL” was used operationally to refer to phenotypically defined CD8-positive T-cell subsets based on CD27/CD45RA expression and does not necessarily indicate confirmed cytotoxic function. According to the manufacturer’s reference data in normal specimens, the relative representation of individual TCR Vβ families varies widely across Vβ families in CD3^+^ and CD3^+^CD8^+^ T-cell subsets; therefore, these categories were used as operational descriptors of Vβ family over-representation and were not intended to indicate functional activation, clonotype identity, or antigen specificity.

Flow cytometric acquisition was performed on a NAVIOS EX flow cytometer (Beckman Coulter, Brea, CA, USA), and data were analyzed using Kaluza Analysis v2.2 (Beckman Coulter, Brea, CA, USA). All monoclonal antibodies are listed in Supplemental Table S1.

### 2.4. Patients

Seven patients with relapsed or refractory TFH-type lymphoma (six AITL and one TFH-NOS) who received at least one cycle of DD at our institution were retrospectively evaluated for treatment response and survival. These 7 patients constituted the DD-treated clinical cohort. Six of these 7 patients, excluding Pt-4, were also included in the lymph node FOXP3 analysis described in Section 2.1. Pt-4 was not included in the FOXP3 analysis because an appropriate lymph node specimen for this analysis was not available. Clinical response after DD was assessed based on available clinical records and imaging findings.

Patient consent for the retrospective clinical and pathological analyses was waived by the institutional ethics board because of the retrospective design of the study. Written informed consent was obtained from all patients who underwent TCR repertoire analysis.

### 2.5. Statistical Analysis

Comparisons of continuous variables between two groups were performed using the two-sided Mann–Whitney U test. Categorical variables were compared using Fisher’s exact test. Because this study was exploratory and involved a limited number of predefined comparisons, no correction for multiple testing was applied. A *p*-value < 0.05 was considered statistically significant. All statistical analyses were conducted using GraphPad Prism 9 (GraphPad Software, San Diego, CA, USA).

The study was approved by the Central Ethical Review Board of the Japanese Red Cross Nagasaki Genbaku Hospital (Approval number: R6-784, Approval date: 4 June 2024) and was conducted in accordance with the principles of the Declaration of Helsinki (1964) and its later amendments.

## 3. Results

### 3.1. Comparison of Intra-Tumoral FOXP3-Positive Cell Density Between TFH-Type and Non-TFH-Type Lymphomas

**Figure 1:**
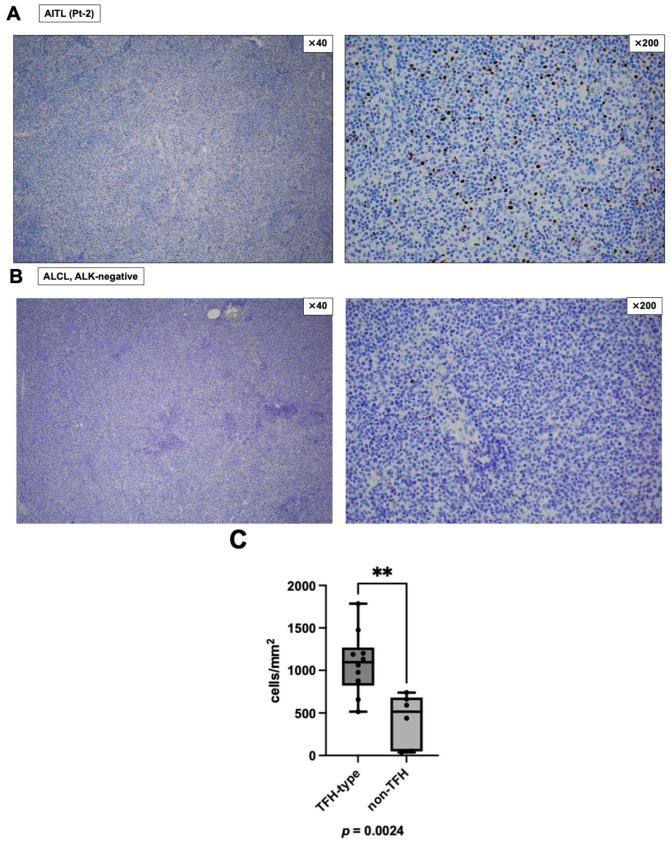
Intra-tumoral FOXP3-positive cell density is higher in TFH-type than non-TFH-type lymphomas. FOXP3-positive mononuclear cells were quantified by IHC and expressed as cells/mm^2^. For each case, 15 intra-tumoral fields were selected in a non-targeted manner to minimize field-selection bias. Hotspot fields were not specifically targeted, but were not intentionally excluded if encountered during field selection. The mean number of FOXP3-positive cells across the 15 fields was used for analysis. (**A**) A representative case of TFH-type lymphoma, angioimmunoblastic T-cell lymphoma (AITL; Pt-2 in Table 2), is shown. Original magnifications of the left and right panels are ×40 and ×200, respectively. (**B**) A representative case of non-TFH-type lymphoma, ALK-negative anaplastic large cell lymphoma (ALCL), is shown. Original magnifications of the left and right panels are ×40 and ×200, respectively. (**C**) Box plots showing FOXP3-positive cell densities in patients with TFH-type lymphoma (n = 10) and non-TFH-type lymphoma (n = 6). Each dot represents one patient. Boxes indicate the median and interquartile range, and whiskers indicate the minimum and maximum values. Groups were compared using the two-sided Mann–Whitney U test. ** *p* < 0.01. Abbreviations: AITL, angioimmunoblastic T-cell lymphoma; ALCL, anaplastic large cell lymphoma; FOXP3, forkhead box P3; IHC, immunohistochemistry; TFH, follicular helper T-cell.

Diagnostic lymph node biopsy samples obtained before systemic chemotherapy from 10 patients with TFH-type lymphoma (seven AITL and three TFH-NOS) and six patients with non-TFH-type lymphoma (two PTCL-NOS, one EBV^+^ nodal NK/T cell lymphoma, one ALK-negative ALCL, and two ATLL) were immunohistochemically stained to quantify FOXP3-positive mononuclear cells (Table 1). Among the 10 TFH-type lymphoma cases, six overlapped with the DD-treated cohort shown in Table 2, excluding Pt-4. The other four TFH-type lymphoma cases had not received DD therapy and were treated with A-CHP, CHOP-like therapy in two cases, or EPOCH. There were no statistically significant differences in sex or age between the TFH-type and non-TFH-type lymphoma groups. FOXP3-positive cell density was significantly higher in TFH-type lymphomas than in the selected non-TFH comparator group (*p* = 0.0024; Figure 1). These findings indicate that TFH-type lymphomas in this cohort had higher intra-tumoral FOXP3-positive cell densities than in the selected non-TFH-type comparator group.

### 3.2. Reduction in Macrophages and FOXP3-Positive Cells in the Skin After DD Treatment

**Figure 2:**
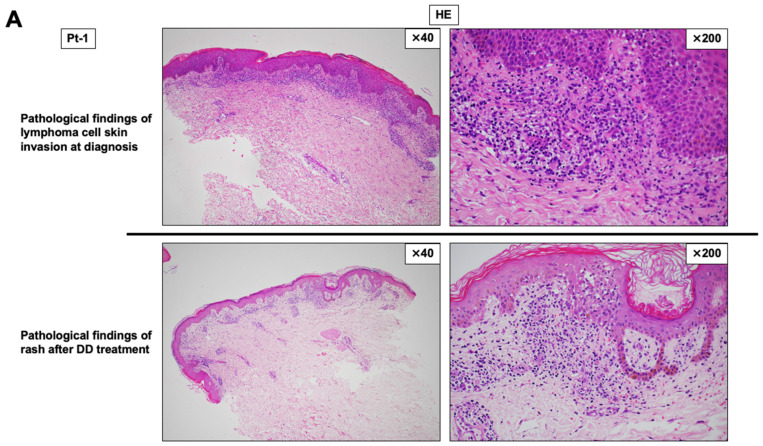

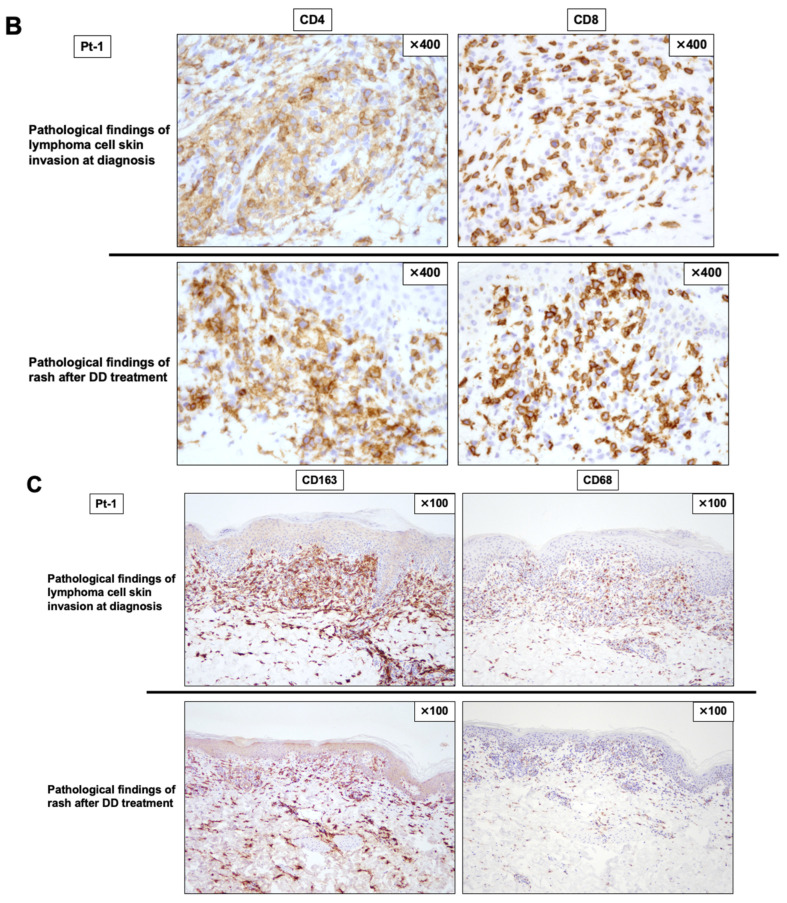

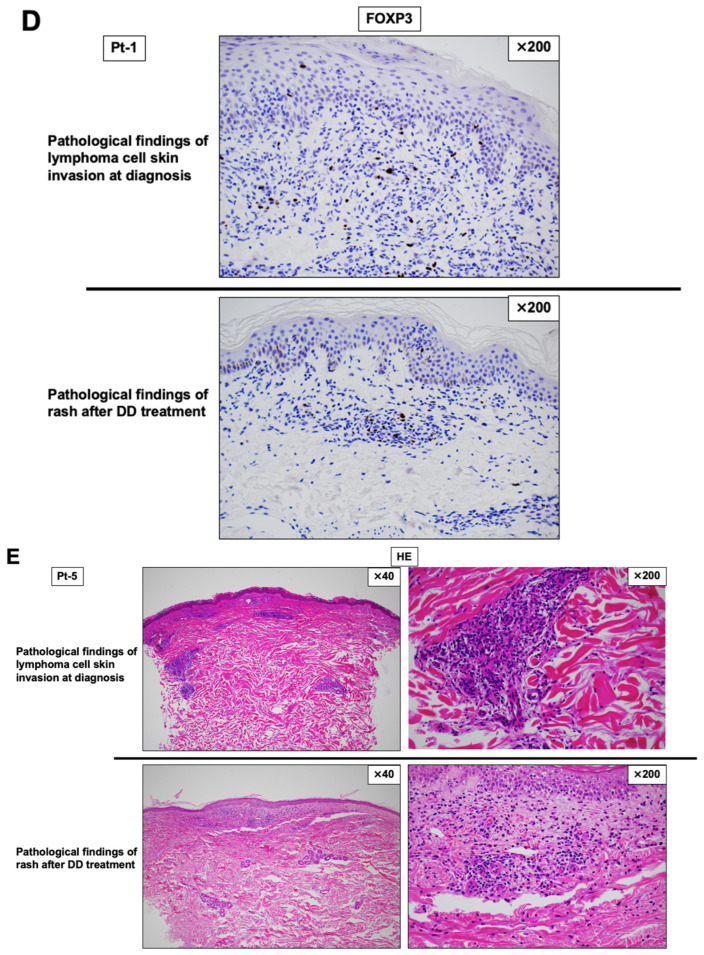

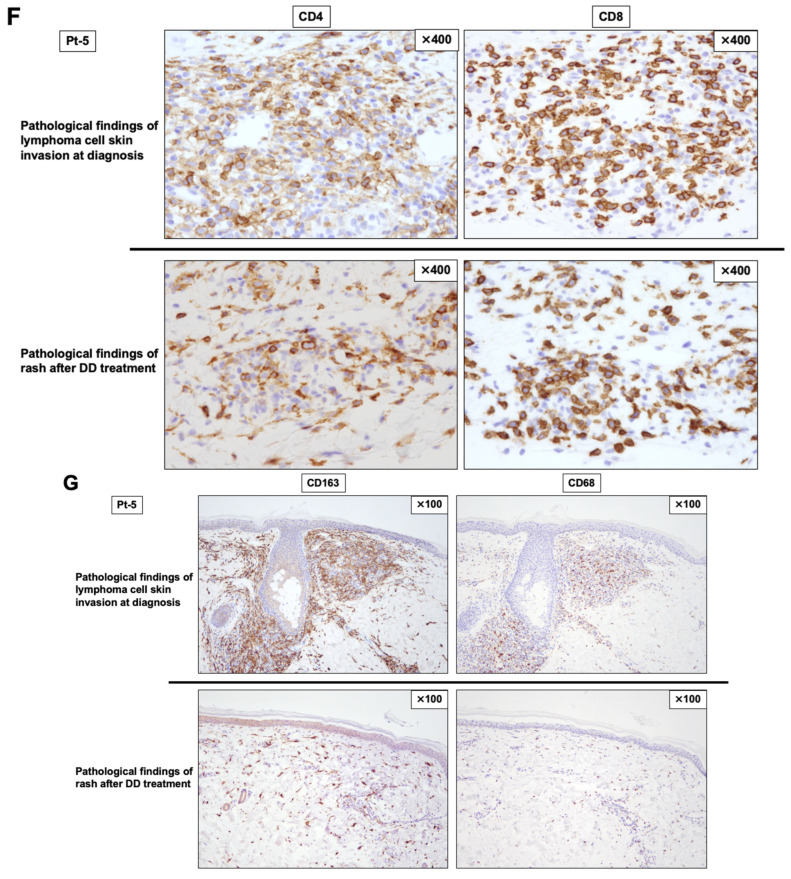

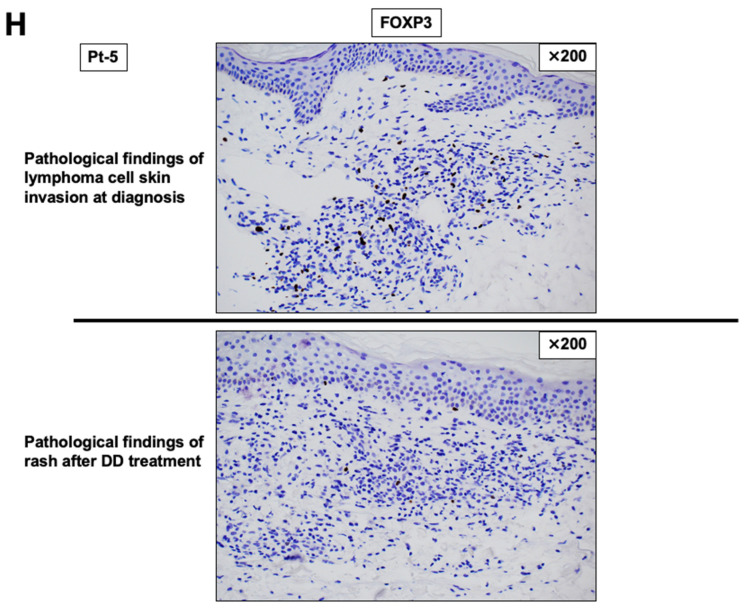
Representative histopathological findings of paired skin biopsy specimens before and after DD therapy in two patients with AITL. Representative histopathological and immunohistochemical findings of cutaneous lymphoma involvement at diagnosis and subsequent skin rash after DD therapy are shown for two patients with AITL: Pt-1 (**A**–**D**) and Pt-5 (**E**–**H**). HE staining showed cutaneous lymphoid infiltration at diagnosis and subsequent inflammatory skin changes after DD therapy (**A**,**E**). In both patients, diagnostic skin biopsy specimens showed prominent CD4-positive atypical lymphoid cells. In post-DD rash biopsy specimens, CD4-positive atypical lymphoid cells appeared less conspicuous, whereas CD8-positive T-cells were relatively increased (**B**,**F**). Immunohistochemistry for CD68 and CD163 showed abundant macrophage/macrophage-rich infiltrates in diagnostic specimens and reduced staining in post-DD rash specimens (**C**,**G**). Representative FOXP3 staining suggested fewer FOXP3-positive cells after DD therapy, although this comparison was qualitative and not based on systematic quantitative image analysis (**D**,**H**). These paired skin biopsy findings should be interpreted as exploratory because the diagnostic specimens represent cutaneous lymphoma involvement, whereas the post-DD specimens were obtained from clinically suspected DD-related skin rash. Abbreviations: AITL, angioimmunoblastic T-cell lymphoma; CD, cluster of differentiation; DD, denileukin diftitox; FOXP3, forkhead box P3; HE, hematoxylin and eosin; IHC, immunohistochemistry. Treatment response and overall survival of seven patients with TFH-type lymphomas treated with DD.

We evaluated paired skin biopsies obtained at diagnosis (with cutaneous lymphoma involvement) and after DD therapy (skin rash suspected of DD-related eruption) in two patients with AITL (Pt-1 and Pt-5; Table 2) (Figure 2). In diagnostic skin biopsies, large atypical lymphoid cells showed CD4-positive phenotype with minimal CD8 positivity, consistent with cutaneous involvement of AITL (Figure 2B,F, upper panels). After DD therapy, HE staining demonstrated decreased inflammatory/atypical cellular infiltration in the epidermis and dermis (Figure 2A,E, lower panels). IHC further showed that CD4-positive large lymphoid cells became scarce, whereas the proportion of CD8-positive T-cells appeared relatively increased in both cases (Figure 2B,F, lower panels). Notably, macrophage markers (CD68 and CD163) were markedly reduced in post-DD skin biopsies compared with diagnostic biopsies (Figure 2C,G). Because CD163 is commonly used as a marker of M2-like macrophages, these findings are consistent with a reduction in macrophage/M2-like macrophage-rich infiltrates following DD therapy. In addition, representative FOXP3 immunostaining of paired skin biopsy specimens suggested a decrease in FOXP3-positive cells after DD therapy in both patients, although the reduction appeared more marked in Pt-5 than Pt-1 (Figure 2D,H). Because the post-DD specimens were obtained from clinically suspected DD-related skin rash rather than residual lymphoma lesions, these findings were interpreted as qualitative and exploratory observations rather than definitive evidence of tumor microenvironment remodeling.

Table 2 summarizes the seven patients with relapsed and/or refractory (R/R) TFH-type lymphoma treated with DD. Six patients had AITL and one patient had TFH-NOS. All patients were older than 70 years, and five were male. Four patients received one line of prior therapy, one received two lines, and two received five lines. DD treatment cycles ranged from 1 to 6. The best treatment response was complete remission (CR) in one patient, partial remission (PR) in five, and progressive disease (PD) in one. Pt-4 achieved CR and had no relapse, but died of COVID-19. Pt-1 achieved PR after one cycle of DD, but DD was discontinued due to rash; forodesine, a purine nucleoside phosphorylase inhibitor, was then initiated and continued for approximately seven months. Pt-1 died of herpes zoster-associated spinal cord infarction without relapse of AITL. Pt-3 achieved PR after two cycles of DD; however, cytokine release syndrome (CRS) occurred and the regimen was changed to romidepsin, a histone deacetylase inhibitor (HDACi). Romidepsin was discontinued due to cytomegalovirus (CMV) infection. Performance status (PS) of Pt-3 deteriorated to four and best supportive care was selected; Pt-3 was transferred to a hospice and died without relapse of AITL. Pt-7 achieved PR after one cycle of DD; however, DD was discontinued because of CRS and CMV infection. Treatment was then switched to tucidinostat, a histone deacetylase inhibitor (HDACi). Because of myelosuppression, tucidinostat could be administered only three times in the first cycle and subsequently discontinued. Nevertheless, the patient maintained PR without further treatment for a prolonged period. After subsequent relapse, DD was readministered for five consecutive days, followed, after a one-week interval, by three weekly doses of tucidinostat, and then a further one-week drug-free interval. This treatment sequence was defined as one cycle and was repeated for three cycles, resulting in transient clinical improvement. However, the lymphoma showed signs of re-progression. Treatment was therefore switched to valemetostat monotherapy, an enhancer of the zeste homolog 1/2 (EZH1/2) inhibitor. Valemetostat induced a response and has been continued thereafter, with PR maintained to date (49 months after the first DD treatment initiation). The overall response rate was 86% in this small retrospective cohort, suggesting potential clinical activity of DD in selected patients with R/R TFH-type lymphoma. However, this finding should be interpreted cautiously given the limited sample size, retrospective design, subsequent therapies, and infectious complications.

### 3.3. Peripheral CD8/TCR Vβ Repertoire Analysis in Two AITL Patients with PR and One AITL Patient with PD Following DD Therapy

**Figure 3:**
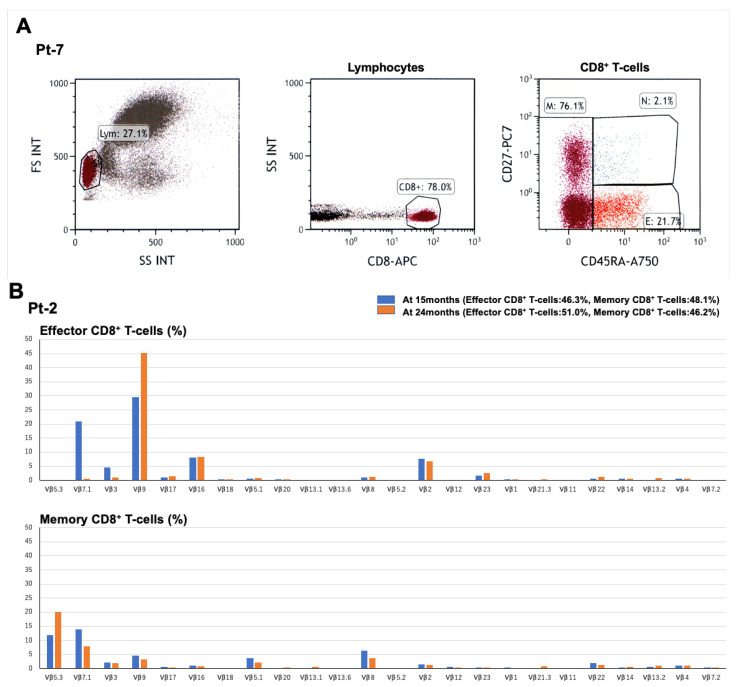

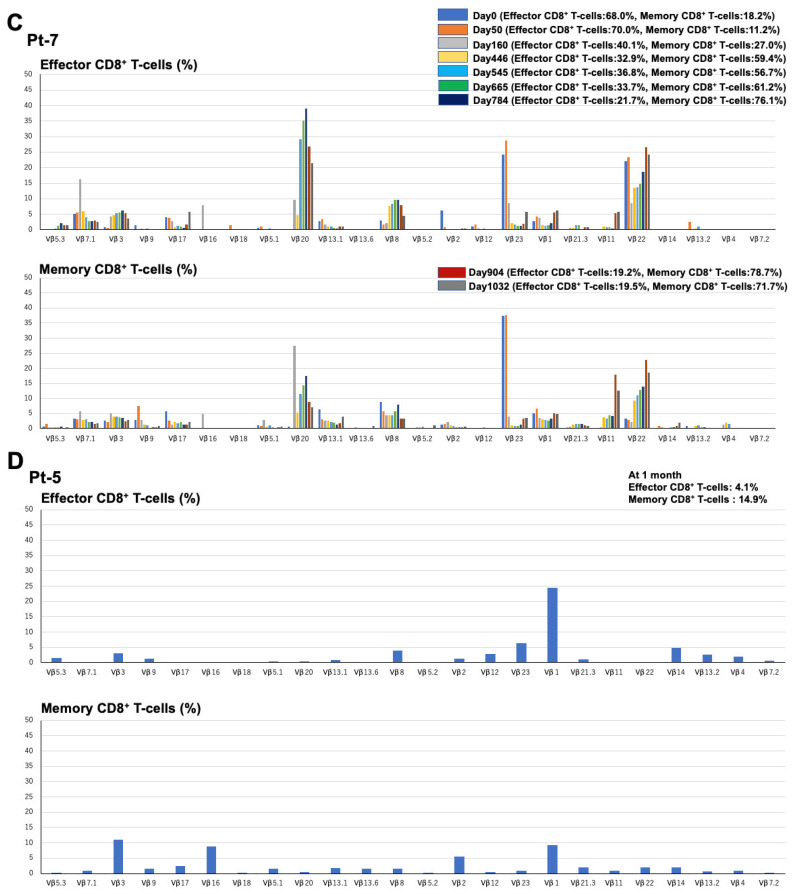
Peripheral CD8/TCR Vβ repertoire patterns in three patients with AITL treated with DD. A representative baseline gating strategy (**A**) and longitudinal changes in TCR Vβ family usage within effector and memory CD8-positive T-cell subsets in Pt-2, Pt-7, and Pt-5 (**B**–**D**) after DD treatment are shown. (**A**) Baseline flow cytometric plots from Pt-7 demonstrating the sequential gating strategy: lymphocyte gating by forward scatter/side scatter, identification of CD8^+^ T-cells, and subdivision into naïve, effector, and memory CD8-positive T-cell subsets using CD27 and CD45RA. (**B**) Longitudinal TCR Vβ patterns in Pt-2 at 15 and 24 months after DD treatment initiation. Vβ9 over-representation was observed in effector CD8-positive T-cells, and Vβ5.3 and Vβ7.1 over-representation was observed in memory CD8-positive T-cells. Additional Vβ16 and Vβ2 over-representation was observed in effector CD8-positive T-cells. (**C**) Longitudinal TCR Vβ patterns in Pt-7 from day 0 to day 1032 after the first DD treatment initiation. Vβ20 and Vβ2.2 over-representation in effector and memory CD8-positive T-cell subsets persisted until relapse on day 851. Vβ2.3 over-representation was evident until day 50 but subsequently decreased. Several Vβ families, including Vβ1, Vβ11, and Vβ2.2, became over-represented after DD retreatment and subsequent treatment modification. (**D**) TCR Vβ patterns in Pt-5 at 1 month after DD treatment initiation. Increased proportions of several Vβ families, including Vβ1, Vβ2.3, Vβ3, Vβ16, and Vβ2, were observed in effector and/or memory CD8-positive T-cell subsets. However, the low total proportions of effector and memory CD8-positive T-cells, 4.1% and 14.9%, respectively, suggest that these changes may not represent meaningful systemic CD8-positive T-cell expansion. A750, Alexa Fluor 750; CD8^+^, proportion of CD8-positive T-cells; DD, denileukin diftitox; E, total proportion of effector CD8-positive T-cells; FS INT, forward scatter intensity; Lym, proportion of lymphocytes; M, total proportion of memory CD8-positive T-cells; N, total proportion of naïve CD8-positive T-cells; PC7, Phycoerythrin-Cy7; SS INT, side scatter intensity; TCR, T-cell receptor; Vβ, variable region beta. All antibodies and IOTest Beta Mark TCR Vβ Repertoire Kit were purchased from Beckman Coulter, Brea, CA, USA.

Three AITL patients treated with DD were analyzed for peripheral CD8-positive T-cell subset distribution and TCR Vβ repertoire using flow cytometry. Peripheral blood mononuclear cells were collected at several time points, and naïve, effector, and memory CD8-positive T-cell subsets were defined using CD8, CD27, and CD45RA [17]. Representative data (Pt-7) are presented in Figure 3A. Naïve, effector, and memory CD8-positive T-cell subsets were defined as CD8^+^CD27^+^CD45RA^+^, CD8^+^CD27^−^CD45RA^+^, and CD8^+^CD27^±^CD45RA^−^, respectively.

Pt-2, who achieved PR following DD therapy, showed a dominant Vβ9-over-represented population within effector CD8-positive T-cells and TCR Vβ5.3/Vβ7.1 over-representation within memory CD8-positive T-cells (Figure 3B). These findings may suggest skewing of multiple CD8-positive T-cell populations with distinct TCR Vβ usage. Pt-7, another DD-responding patient, also showed Vβ over-representation in several effector and memory CD8-positive T-cell populations (Figure 3C).

Pt-7 maintained a sustained clinical response for a prolonged period after DD-based therapy followed by tucidinostat administration. However, relapse of AITL was confirmed by lymph node biopsy on day 851 after DD treatment initiation. Vβ20 and Vβ2.2 over-representation in effector and memory CD8-positive T-cell subsets persisted until relapse and remained detectable after relapse. In contrast, Vβ2.3 over-representation, which was evident until day 50, subsequently decreased. After subsequent treatment modification, including switching to valemetostat, the patient achieved a response and has maintained PR to date. Several Vβ families, including Vβ1, Vβ11, and Vβ2.2, became over-represented during subsequent treatment after relapse.

In contrast to the two responding patients, Pt-5 showed resistance to DD therapy. At 1 month after DD treatment, TCR repertoire analysis showed increased proportions of several Vβ families, including Vβ1, Vβ2.3, Vβ3, Vβ16, and Vβ2, within effector and/or memory CD8-positive T-cell subsets. However, the total proportions of effector and memory CD8-positive T-cells were low, at 4.1% and 14.9%, respectively (Figure 3D). These findings suggest that, although Vβ skewing was observed, it may not represent functionally or clinically meaningful systemic CD8-positive T-cell expansion in this particular patient.

## 4. Discussion

PTCLs remain difficult to treat, particularly in older patients and those with relapsed/refractory disease. In this study, we investigated TFH-type lymphomas treated with DD and provide clinical and translational observations suggesting that DD may influence not only tumor burden but also the immunologic contexture of the tumor microenvironment (TME).

A principal finding is that TFH-type lymphomas (AITL and TFH-NOS) exhibited significantly higher intra-tumoral densities of FOXP3-positive cells compared with non-TFH-type T-cell lymphomas (Table 1 and Figure 1). TFH-type lymphomas are characterized by a complex immune-rich milieu, and our data support the notion that an immunosuppressive compartment—represented at least in part by FOXP3-positive mononuclear cells—is prominent in these lymphomas. Recent studies have also emphasized the complex immune microenvironment of AITL and the potential clinical relevance of FOXP3-positive cells. Liu et al. analyzed 46 AITL cases using digital pathology and reported that low intra-tumoral FOXP3 expression was associated with aggressive clinical features and worse progression-free survival, supporting the biological and prognostic relevance of FOXP3-positive cells in AITL [18]. In addition, a recent overview of AITL highlighted that the tumor microenvironment is composed of multiple immune and stromal components and is closely linked to AITL pathogenesis and therapeutic development [19]. Therefore, our findings should be interpreted not as establishing FOXP3-positive cells as functionally suppressive Tregs, but as supporting the presence of a FOXP3-enriched immune microenvironment in TFH-type lymphoma. From a therapeutic perspective, these results provide a biologic rationale for approaches that target or modulate immune cell populations in TFH-type lymphoma.

In paired skin biopsies from two AITL patients (Pt-1 and Pt-5), we observed a marked reduction in macrophage-associated markers after DD therapy (Figure 2C,G). Specifically, CD68 and CD163 staining was markedly reduced in post-DD biopsies compared with diagnostic biopsies. Because CD68 is a pan-macrophage marker and CD163 is commonly used as a marker of M2-like macrophages [20,21,22], these findings are consistent with a reduction in macrophage/M2-like macrophage-rich infiltrates following DD therapy. In addition, representative FOXP3 immunostaining suggested a decrease in FOXP3-positive cells after DD therapy in both patients, although the reduction appeared more marked in Pt-5 than Pt-1 (Figure 2D,H). Importantly, these tissue-based observations were made in skin rather than nodal tissue and were not based on systematic quantitative image analysis; therefore, they should be interpreted as hypothesis-generating. Nevertheless, the concordant decrease in macrophage markers and the suggested decrease in FOXP3-positive cells support the possibility that DD treatment may be associated with changes in multiple immunosuppressive compartments in clinical specimens.

We further explored whether DD therapy was accompanied by changes in peripheral CD8-positive T-cell profiles using longitudinal flow cytometric TCR Vβ repertoire analysis in three AITL patients. In two responders (Pt-2 and Pt-7), selective over-representation of several Vβ families was observed in effector and/or memory CD8-positive T-cell subsets (Figure 3B,C). In contrast, in the non-responder (Pt-5), convincing systemic expansion of effector/memory CD8-positive T-cell subsets was not observed (Figure 3D). Although flow cytometric Vβ analysis cannot establish clonotype identity, antigen specificity, or cytotoxic function, these observations suggest that clinical responses to DD-based therapy can be accompanied by peripheral CD8/TCR Vβ skewing in some cases. The dynamic changes in Vβ family usage over time in Pt-7 further suggest that peripheral CD8-positive T-cell profiles may be heterogeneous and may change during the clinical course.

In addition to the dynamic changes in individual Vβ families, Pt-7 also showed an intriguing shift in the overall composition of CD8-positive T-cell subsets during disease control, with a decrease in the proportion of total effector CD8-positive T-cells and a reciprocal increase in total memory CD8-positive T-cells over time. Although this observation is based on a single patient and should therefore be interpreted cautiously, it raises the possibility that an increased total memory CD8-positive T-cell proportion may represent one immunologic correlate of tumor reduction or sustained disease control. Interestingly, this pattern is in line with our previous observation in chronic myeloid leukemia, in which patients with long-lasting treatment-free remission tended to show an increased proportion of memory CD8-positive T-cells [23]. Taken together, these findings may suggest that reduction in tumor burden can be accompanied by the relative enrichment of memory CD8-positive T-cells, at least in some hematologic malignancies.

By contrast, Pt-5 did not show convincing systemic expansion patterns in effector or memory CD8-positive T-cell subsets, despite tissue-based findings being consistent with a reduction in macrophage/M2-like macrophage-rich infiltrates and a suggested decrease in FOXP3-positive cells after DD therapy. This discrepancy may be informative. In tumor immunity, reduction in immunosuppressive cells is likely important, but it may not by itself be sufficient to achieve meaningful antitumor effects in refractory disease unless accompanied by systemic CD8-positive T-cell changes that are sufficient to support antitumor immunity. In other words, our findings raise the possibility that both components may need to be considered when interpreting clinically relevant tumor control. These observations suggest that changes in tissue immune infiltrates and peripheral CD8/TCR Vβ profiles may occur in association with clinical responses in some patients. However, the present data do not establish DD-mediated immune remodeling, antitumor immunity, or functional CD8-positive T-cell responses. Therefore, these findings should be interpreted as exploratory immune correlates rather than evidence of a mechanistic model.

Mechanistically, DD targets the IL-2R pathway. Tregs frequently express high levels of IL-2Rα, providing a plausible basis for their susceptibility to IL-2R-directed approaches. In addition, IL-2R expression has been reported on subsets of activated immune cells [24], raising the possibility that DD may influence multiple immune populations within the TME. In this framework, our findings are compatible with the possibility that DD treatment may be accompanied by immune changes in addition to its known direct cytotoxic activity against IL-2R-expressing cells. However, the present data do not determine whether such immune changes are a primary mechanism of response or secondary to tumor reduction and other time-dependent clinical factors.

Clinically, DD showed potential activity in this elderly cohort, although interpretation is limited by the small sample size, retrospective design, subsequent therapies, and infectious complications. In a previous multicenter phase II study of E7777 in Japanese patients with relapsed or refractory PTCL and CTCL, the objective response rate was 36% among patients with PTCL/CTCL, with manageable safety at 9 mg/kg/day [15]. Our study differs from that trial in that it focused specifically on TFH-type lymphoma and incorporated exploratory tissue and peripheral immune profiling. These observations suggest that DD-based therapy may warrant further evaluation in selected patients with R/R TFH-type lymphoma, rather than establishing its efficacy in this setting. Our translational observations also raise a testable hypothesis that DD could be integrated into treatment sequences aimed at modifying the immune milieu, potentially enhancing the effectiveness or durability of subsequent therapies with different mechanisms of action. Such sequencing concepts should be evaluated prospectively.

This study has limitations. The cohort was small and derived from a single institution, limiting generalizability. The non-TFH-type comparator group was heterogeneous and included PTCL-NOS, EBV-positive nodal NK/T-cell lymphoma, ALK-negative ALCL, and ATLL. Therefore, this comparison should be interpreted as an exploratory comparison against selected non-TFH-type T-cell lymphoma comparators rather than as a definitive disease-matched analysis. The paired tissue analysis was restricted to skin biopsies and did not include systematic quantification across multiple fields or whole-slide analysis. The Vβ repertoire approach provides an operational measure of Vβ over-representation but cannot define tumor specificity or CTL function. Moreover, because this was an observational study, the impact of concomitant or subsequent therapies and time-dependent clinical factors cannot be fully disentangled.

## 5. Conclusions

In summary, TFH-type lymphomas showed significantly higher intra-tumoral FOXP3-positive cell density than selected non-TFH-type lymphoma comparators. In two paired skin biopsy cases, post-DD rash specimens showed reduced CD68 and CD163 staining and fewer FOXP3-positive cells compared with diagnostic skin lymphoma specimens. In responding cases, peripheral CD8-positive T-cell subsets showed TCR Vβ skewing. These findings are exploratory and do not establish a causal mechanism, but they support further prospective studies incorporating quantitative tissue analysis, TCR sequencing, and functional immune assays to clarify correlates of DD treatment in TFH-type lymphoma.

## Figures and Tables

**Table 1:** Summary of patients analyzed regarding FOXP3-positive cell density in lymphoma tissues.

	TFH-Type Lymphoma (*n* = 10) AITL: *n* = 7 TFH-NOS: *n* = 3	Non-TFH Lymphoma (n = 6) PTCL-NOS: *n* = 2 EBV^+^ Nodal NK/T: *n* = 1 ALK^−^ ALCL: *n* = 1 ATLL: *n* = 2	*p* Value
Male/female	4/6	2/4	>0.9999
Age	72–93	62–89	0.3820
(median)	(79)	(78)	
FOXP3-positive cells (/mm^2^)	516–1786	36–89	0.0024
(median)	(1121)	(51)	

All lymph node specimens were diagnostic biopsy samples obtained before systemic chemotherapy. Among the 10 TFH-type lymphoma cases analyzed for FOXP3-positive cell density, 6 overlapped with the DD-treated cohort shown in Table 2, excluding Pt-4; the remaining 4 cases had not received DD therapy. Abbreviations: AITL, angioimmunoblastic T-cell lymphoma; ALK^−^ ALCL, ALK-negative anaplastic large cell lymphoma; ATLL, adult T-cell leukemia/lymphoma; EBV^+^ nodal NK/T, Epstein–Barr virus-positive nodal natural killer/T-cell lymphoma; PTCL-NOS, peripheral T-cell lymphoma not otherwise specified; TFH, follicular helper T-cell.

**Table 2:** Summary of DD therapy for TFH-type lymphoma.

ID	Age at 1st DD	Histology	Treatment Before DD	Number of DD Treatment	Best Response of DD	Treatment After DD	TTNT or Death from DD Initiation (Months)	OS from DD Initiation (Months)	CD8/TCR Vβ Skewing
Pt-1	73	AITL	Moga-EPOCH	1	PR	Forodesine (No relapse)	1.6	14.4	not examined
Pt-2	83	AITL	Moga-EPOCH Romidepsin BV Forodesine GCD	3	PR	Romidepsin Pralatrexate A-CHP Darinaparsin	2.2	25.9	Effector CD8^+^ T-cells: +++ Memory CD8^+^ T-cells: +++
Pt-3	77	AITL	A-CHP	2	PR	Romidepsin (No relapse)	1.6	7.2	not examined
Pt-4	79	AITL	THP-COP GDP GCD Romidepsin Pralatrexate	5	CR	(No relapse)	13.9	13.9	not examined
Pt-5	80	AITL	Moga-EPOCH	1	PD	BV	2.1	3.1	Effector CD8^+^ T-cells: − Memory CD8^+^ T-cells: −
Pt-6	73	TFH-NOS	A-CHP BV	6	PR	BV Romidepsin Moga-EPOCH A-CHP	5.5	14	not examined
Pt-7	83	AITL	A-CHP	1	PR	Tucidinostat after relapse DD/tucidinostat Valemetostat	1.9	49	Effector CD8^+^ T-cells: +++ Memory CD8^+^ T-cells: +++

“+++” indicates prominent TCR Vβ over-representation in effector and/or memory CD8-positive T-cell subsets; “−” indicates no convincing TCR Vβ over-representation. These findings do not establish clonotype identity, antigen specificity, or cytotoxic function. A-CHP: combination chemotherapy composed of brentuximab vedotin, cyclophosphamide, doxorubicin hydrochloride, and prednisolone; AITL, angioimmunoblastic T-cell lymphoma; DD, denileukin diftitox; EPOCH, combination chemotherapy composed of etoposide, prednisolone, vincristine, cyclophosphamide, and doxorubicin hydrochloride; ID, identification; Moga, mogamulizumab; OS, overall survival; TCR, T-cell receptor; TFH-NOS, follicular helper T-cell lymphoma not otherwise specified; THP-COP, combination chemotherapy composed of pirarubicin, cyclophosphamide, vincristine, and prednisolone; TTNT, time to next treatment.

## Data Availability

The data supporting the findings of this study are available from the corresponding author upon reasonable request. These data are not publicly available because they contain information that could compromise patient privacy and/or are subject to ethical restrictions.

## Supplementary Materials

The following supporting information can be downloaded at https://www.mdpi.com/article/10.3390/cancers18101529/s1, Table S1: Immunohistochemistry and flow cytometry antibody panel.
