# Supplementary material for: Immune Correlates of Denileukin Diftitox Treatment in TFH-Type Lymphoma
Source: Cancers (Basel). 2026 May 9;18(10):1529. doi: 10.3390/cancers18101529 (PMC13204862; doi:10.3390/cancers18101529)
Supplement: Supplementary file 1 [file cancers-18-01529-s001.zip › cancers-4253678-supplementary.pdf]

Supplemental Table S1. Immunohistochemistry and flow cytometry antibody panel

Assay	Marker/Reagent	Chromogen/ Fluorochrome	Clone name	Catalog number	Manufacturer
Immunohistochemistry					
	FOXP3	DAB	236A/E7	ab2034	Abcam
	CD4	DAB	4B12	IR649	Dako
	CD8	DAB	C8/144B	IR623	Dako
	CD68	DAB	kp-1	IR609	Dako
	CD163	DAB	10D6	PA0090	Leica Biosystems
Flow cytometry					
	CD8	APC	B9.B11	IM2469	Beckman Coulter
	CD27	PC7	A4CD27	A54823	Beckman Coulter
	CD45RA	ECD	2H4LD11LDB9 (2H4)	IM2711U	Beckman Coulter
	IOTest Beta Mark TCR Vβ Repertoire Kit	–	–	IM3497	Beckman Coulter

For immunohistochemistry, antibody for FOXP3 was purchased from Abcam, Waltham, MA, USA. Antibodies for CD4, CD8, and CD68 were purchased from Dako, Carpinteria, CA, USA, and antibody for CD163 was purchased from Leica Biosystems, Buffalo Grove, IL, USA. For flow cytometry, all antibodies and IO Test Beta Mark TCR Repertoire Kit were purchased from Beckman Coulter, Brea, CA, USA.

CD, cluster of differentiation; APC, allophycocyanin; ECD, phycoerythrin-Texas Red; DAB, 3,3'-Diaminobenzidine; PC7, Phycoerythrin-Cy.
